# Disentangling the Possible Drivers of *Indri indri* Microbiome: A Threatened Lemur Species of Madagascar

**DOI:** 10.3389/fmicb.2021.668274

**Published:** 2021-08-06

**Authors:** Federico Correa, Valeria Torti, Caterina Spiezio, Alice Checcucci, Monica Modesto, Luigimaria Borruso, Luciano Cavani, Tanja Mimmo, Stefano Cesco, Diana Luise, Rose M. Randrianarison, Marco Gamba, Nianja J. Rarojoson, Maurizio Sanguinetti, Maura Di Vito, Francesca Bugli, Paola Mattarelli, Paolo Trevisi, Cristina Giacoma, Camillo Sandri

**Affiliations:** ^1^Department of Agricultural and Food Sciences, University of Bologna, Bologna, Italy; ^2^Department of Life Sciences and Systems Biology, University of Torino, Turin, Italy; ^3^Department of Animal Health Care and Management, Parco Natura Viva – Garda Zoological Park, Verona, Italy; ^4^Faculty of Science and Technology, Free University of Bolzano-Bozen, Bolzano, Italy; ^5^Groupe d’Étude et de Recherche sur les Primates de Madagascar, Antananarivo, Madagascar; ^6^Mention d’Anthropobiologie et de Deìveloppement Durable, Université de Antananarivo, Antananarivo, Madagascar; ^7^Laboratoire de Pédologie, FOFIFA à Tsimbazaza, Antananarivo, Madagascar; ^8^Dipartimento di Scienze Biotecnologiche di Base, Cliniche Intensivologiche e Perioperatorie, Università Cattolica del Sacro Cuore, Rome, Italy; ^9^Dipartimento di Scienze di Laboratorio e Infettivologiche, Fondazione Policlinico Universitario A. Gemelli IRCCS, Rome, Italy

**Keywords:** gut microbiome, soil quality, non-human primate, animal ecology, endangered species, geophagy, forest ecology

## Abstract

Research on the gut microbiome may help with increasing our understanding of primate health with species’ ecology, evolution, and behavior. In particular, microbiome-related information has the potential to clarify ecology issues, providing knowledge in support of wild primates conservation and their associated habitats. Indri (*Indri indri*) is the largest extant living lemur of Madagascar. This species is classified as “critically endangered” by the IUCN Red List of Threatened Species, representing one of the world’s 25 most endangered primates. Indris diet is mainly folivorous, but these primates frequently and voluntarily engage in geophagy. Indris have never been successfully bred under human care, suggesting that some behavioral and/or ecological factors are still not considered from the *ex situ* conservation protocols. Here, we explored gut microbiome composition of 18 indris belonging to 5 different family groups. The most represented phyla were Proteobacteria 40.1 ± 9.5%, Bacteroidetes 28.7 ± 2.8%, Synergistetes 16.7 ± 4.5%, and Firmicutes 11.1 ± 1.9%. Further, our results revealed that bacterial alpha and beta diversity were influenced by indri family group and sex. In addition, we investigated the chemical composition of geophagic soil to explore the possible ecological value of soil as a nutrient supply. The quite acidic pH and high levels of secondary oxide-hydroxides of the soils could play a role in the folivorous diet’s gut detoxification activity. In addition, the high contents of iron and manganese found the soils could act as micronutrients in the indris’ diet. Nevertheless, the concentration of a few elements (i.e., calcium, sulfur, boron, nickel, sodium, and chromium) was higher in non-geophagic than in geophagic soils. In conclusion, the data presented herein provide a baseline for outlining some possible drivers responsible for the gut microbiome diversity in indris, thus laying the foundations for developing further strategies involved in indris’ conservation.

## Introduction

Studies on human and animal microbiome have provided compelling evidence that gut microbial diversity is fundamental in shaping metabolic and regulatory networks involved in the maintenance of host healthy status, as well as in a spectrum of disease states (Shreiner et al., 2015; Sandri et al., 2020). Indeed, the mammalian gut microbiome plays a crucial role in host physiology, supporting vitamin synthesis, helping in complex carbohydrates digestion, toxins metabolism, pathogens antagonism, and immune system modulation (Cresci and Bawden, 2015). Factors influencing the differences in mammalian gut microbiome are debated: host behaviors and environments, biogeography, and host genetic effects (e.g., gastrointestinal tract morphology) are of great importance (Lankau et al., 2012; Moeller et al., 2013; Amato et al., 2016). Previous studies showed that frequent social networks are positively associated with high similarity in gut microbial diversity (Tung et al., 2015; Perofsky et al., 2019). Vertical transmission from parent to offspring is the first driver for gut microbiome development, but horizontal transmission from the environment provides a crucial microbial colonization route. Even if microbial transmission due to sociality has traditionally been viewed as a risk for pathogen exposure, it may also be essential to host health. Therefore, it can avoid bottleneck-induced extinctions that could occur when the transmission of microorganisms is strict from parent to offspring. Indeed, it can allow the acquisition of beneficial microbes, particularly those that might not be gained through vertical transmission (Lombardo, 2008; Amaral et al., 2017). Moeller et al. (2013) underlined that gut microbial populations’ social inheritance might be fundamental for preserving microbial diversity over evolutionary time scales.

The lemurs harbored species-specific and/or populations specific microbiomes, which are mainly influenced by their dietary specificity, even on a seasonal basis (Fogel, 2015; Greene et al., 2020). Globally, host habitat is one of the most important factors for gut microbiome modulation, and recently, increasing attention has been devoted to the soil. Indeed, a recent study (Grieneisen et al., 2019) on the gut microbiome of terrestrially living baboons showed that bare soil exceeds 15 times the predictive ability of host genetics in shaping the gut microbiome. Studies in mice (Li et al., 2016; Zhou et al., 2018) confirmed that the effect of soil on gut microbiome composition is comparable to that exerted by diet. Therefore, these studies suggest that contact/ingestion of soil components is beneficial for a healthy gut microbiome.

**FIGURE 1:**
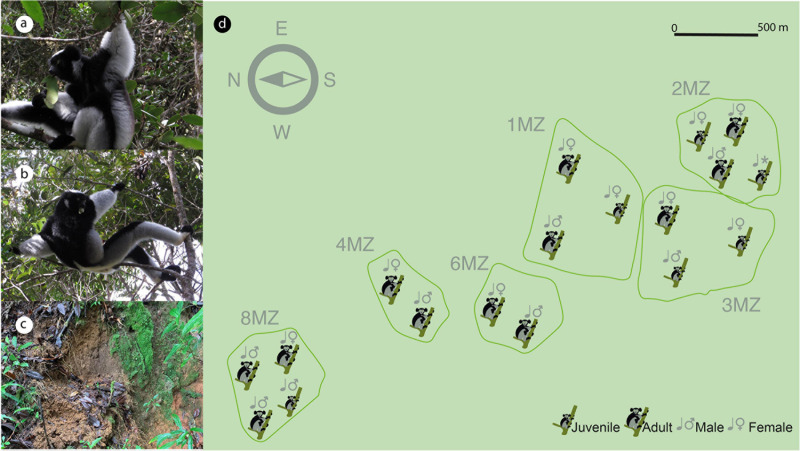
Distribution of the territories occupied by the indri family groups sampled (main figure, **d**) and composition of each single group. Both adult and youngster indris, both sexes, feed on leaves **(a,b)** and perform geophagic behavior, eating soil in specific sites **(c)**.

*Indri indri* is the largest extant living lemur (Figure 1 and Supplementary Video 1). It is mainly arboreal and is the only lemur that communicates using songs. Indris songs mediate both intra- and inter-group communication (Torti et al., 2013) and relay information regarding individual features (i.e., sex and age) (De Gregorio et al., 2019, 2021). This species has never successfully been kept in a controlled environment and it is considered one of the Malagasy most critically endangered lemurs according to the IUCN Red List of Threatened Species (King et al., 2020), representing one of the world’s 25 most endangered primates (Torti et al., 2019). This species is also listed in Appendix I of CITES (Heinen and Mehta, 1999). Indris are territorial, socially primates living in small family groups (Pollock, 1979; Bonadonna et al., 2019), generally consisting of an adult male and female with their related offspring (2–6 individuals) (Torti et al., 2013; Gamba et al., 2016).

Non-human primates are characterized by many dietary specializations (Campbell, 2017). In particular, the ability to consume leaves is typical of new world monkeys (e.g., howler monkeys), old world monkeys (e.g., colobines), apes (e.g., gorillas), and also prosimians (e.g., indris, bamboo lemurs, and sportive lemurs). Indri is the most specialized folivorous among lemurs and, as such, has the highest degree of morphological specialization for leaves’ consumption and digestion. Leaves contain carbohydrates, including cellulose and hemicellulose, and secondary metabolites, including toxic ones such as tannins and phenolics (Norconk et al., 2009). Indris are characterized by the typical morphology and anatomical specializations of folivorous primates, such as hypertrophic salivary glands, voluminous stomachs, sacculated caeca, and looped colons that facilitate efficient fermentation of leaf matter (Greene et al., 2020). The species shows a preference for immature leaves (72%) with a reduced emphasis on fruit seeds/whole fruits (16%) and flowers (7%) (Powzyk, 1997). Leaves and fruit seeds could contain toxic compounds varying in percentage depending on the season, maturity, etc. (Pebsworth et al., 2019). In addition, indris perform geophagy by consuming soil intentionally (Britt et al., 2002; Borruso et al., 2021). Some evidence suggests that geophagy is an adaptive behavior to protect from ingested toxic compounds and mineral supplementation as it facilitates consumption of plants binding toxic plant secondary compounds (PSCs) (Pebsworth et al., 2019). As a result of metabolic activity, plants with relevant antioxidant properties produce primary and secondary compounds. Nevertheless, several metabolites are universally distributed in many plant species; some are unique to individual plant cultivars and fill essential functions (Geilfus, 2019).

Studies regarding geophagy across non-human primates revealed that they eat items high in PSCs. Furthermore, they consume soil more often than sympatric populations, suggesting a decrease in gastrointestinal distress caused by PSCs. Geophagy can help the utilization of dietary resources high in PSCs, expanding the range of dietary components (Overdorff, 1993; Bocian, 1997; Powzyk and Mowry, 2003; Dew, 2005; Pebsworth et al., 2019). In addition to dietary toxins, mineral deficiencies, diarrhea, and altered gut pH were reported to cause geophagy (Krishnamani and Mahaney, 2000; Ferrari et al., 2008; Young et al., 2011). As these processes are not necessarily mutually exclusive, geophagy can play different functions, such as rare element supplementation, detoxification, and protection (Davies and Baillie, 1988; Huffman et al., 1997; Krishnamani and Mahaney, 2000; Pebsworth et al., 2019). Interestingly, geophagic soil could also be a reservoir for microbial species affecting indris’ gut microbiome (Borruso et al., 2021). The highly specialized diet, physiology, and morphology of indri’s gut may contribute to their susceptibility in a human-controlled environment. This is in analogy for what has been described for other endangered folivorous primate whose breeding was unsuccessful (Hale et al., 2018, 2019).

Understanding the drivers of the gut indris microbiome and their relationship to the soil could be essential for planning strategies to conserve, monitor, and promote their health. Whether the gut microbiome facilitates the use of these hard-to-digest food items, it would be crucial to characterize the bacterial gut microbiome’s shaping factors. Therefore, our work aimed to analyze: (1) the gut microbiome composition of wild indris belonging to five different familiar groups in Maromizaha, eastern Madagascar; (2) the potential drivers affecting host-microbial diversity, including sex, family group, and age class (3) the chemical composition of geophagic and non-geophagic soil, to unravel the possible adaptive ecological value as nutrient supply.

## Materials and Methods

### Fecal and Soil Samples Collection

Fecal and soil samples were collected in a very narrow temporal window (between December 4th and 6th, 2018) to avoid confounding potential seasonal effects. Individual fecal samples were obtained from 18 indris (fecal material) belonging to 5 different social family groups (Table 1 and Figure 1) (latitude 18°57′S and 19°00′S, longitude 48°26′E and 48°31′E, Madagascar). The samples were collected immediately after defecation, when only one animal, recognized using natural marks (Torti et al., 2013), was present. This procedure was essential to avoid individual misidentification during the sampling process (Bonadonna et al., 2019). Approximately 5 g of fecal samples were collected from each of the 18 individuals (Table 1) following the procedure described in Borruso et al. (2021).

**TABLE 1:** Description of each indri individual including sex, class age (Adult, >6 years; Juvenile, <6 years; NA*, not available) and family group, bacterial observed richness, and bacterial Shannon index values.

**Samples ID**	**Sex**	**Class age**	**Family group**	**Observed richness**	**Shannon**
L	Female	Adult	1MZ	44	2.87
M	Female	Juvenile	1MZ	41	2.55
O2	Male	Adult	1MZ	43	2.11
R	Female	Juvenile	2MZ	44	2.55
N2	NA*	Juvenile	2MZ	44	2.76
P	Male	Adult	2MZ	46	2.67
Q	Female	Adult	2MZ	47	2.90
G	Female	Juvenile	3MZ	35	2.55
H	Male	Juvenile	3MZ	37	1.95
I	Female	Adult	3MZ	35	2.41
C	Female	Adult	4MZ	38	2.70
E2	Male	Adult	4MZ	45	2.40
K	Female	Adult	6MZ	47	2.75
S	Male	Adult	6MZ	39	2.59
A2	Female	Adult	8MZ	55	2.94
B2	Male	Juvenile	8MZ	58	2.69
D2	Male	Adult	8MZ	55	2.62
F2	Male	Adult	8MZ	58	2.89

Each sample was classified according to the following categories: sex, family group, and age class (Adult > 6 years and Juvenile < 6 years) (Table 1 and Figure 1). In addition, soil samples were collected from seven geophagic and seven non-geophagic (control) sites. All the geophagic sites were at the bases of trees uprooted by wind or rainfall, with the lower soil horizons exposed. We noted the location (waypoint) during soil-eating events, and we followed behaviors before and after the geophagy event. Control sites were selected from areas with the same characteristics (slope, vegetation, etc.) and located at less than 20 m from geophagic sites after removing the superficial soil layer to sample the same soil layer of the geophagic sites. The presence of the superficial layer together with debris proved that the groups have never used the control locations to consume soil. All samples were maintained in a portable cooler with ice packs before arrival at the lab.

### Soil Characterization

Soil samples were air-dried, milled, and sieved at 2 mm for soil analysis in agreement with Soil Science Society of America (SSSA) methods (Sparks et al., 1996). Briefly, pH was determined in water (1:2.5, m/V), total carbon (C), and total nitrogen (N) using an elemental analyzer (CHNS-O Elemental Analyzer 1110, Thermo Scientific GmbH, Germany). Pseudo total element concentrations were determined after acid mineralization with aqua regia and hydrogen peroxide in an Ethos TC microwave lab station (Milestone, Italy) using an inductively coupled plasma optical emission spectrometer (ICP-OES, Ametek Spectro, Arcos, Germany). Available metals were determined by ICP-OES after extraction for 2 h with 1 mol L^–1^ ammonium nitrate (NH_4_NO_3_) solution (1:2.5, m/V).

### DNA Extraction and NGS Sequencing

Total DNA was isolated and extracted from indri fecal samples with DNeasy PowerSoil Kit (QIAGEN, Hilden, Germany) with slight modifications. Briefly, the lysis step was enhanced using a bead-beater (FastPrep 24G, MP Biomedicals, France), in which the “Powerbead” tubes containing the pellets (250 mg of fecal sample) and 800 μL of CD1 solution were subjected to two cycles of bead-beating at a speed of 4 m/s for 60 s with 45 s pause between cycles. The final elution volume was 100 μL in water. DNA was checked for purity (absorbance ratio 260/280 and 260/230) by spectrophotometry using NanoDrop (Fisher Scientific, 13 Schwerte, Germany) and quantified with the fluorometer Qubit^®^ 2.0 (Invitrogen, Italy). Next, the DNA concentration of each sample was normalized to 1 ng μL^–1^. The PCR was performed amplifying the V3–V4 region of the 16S rRNA gene (∼460 bp) with the primers Pro341F (5′-TCGTCGGCAGCGTCAGATGTGTATAAGAGACAGCCTAC GGGNBGCASCAG-3′) and Pro805R (5′-GTCTCGTGGGCTCG GAGATGTGTATAAGAGACAGGACTACNVGGGTATCTAAT CC-3′) (Takahashi et al., 2014), using Platinum Taq DNA Polymerase High Fidelity (Thermo Fisher Scientific, Italy). The thermal cycling protocol consisted of the following conditions: initial denaturation at 94°C for 1′, followed by 25 cycles of denaturation at 94°C for 30″, annealing at 55°C for 30″, and extension 65°C for 45″, ending with 1 cycle at 68°C for 7′. Further, PCR samples were sent to BMR-Genomics Ltd., that according to the standard protocols carried out the other steps of the workflow and finally sequenced the libraries using a MiSq platform (300 × 2 bp) (Illumina Inc., San Diego, CA, United States).

The raw reads obtained are publicly available at the Sequence Read Archive (SRA) under the accession number: PRJNA701813.

### Bioinformatic Analysis

Sequencing data analysis was performed using DADA2 1.14.0 (Callahan et al., 2016) running on R 3.6.2 (R Core Team, 2021). The forward and reverse reads were trimmed to remove low-quality nucleotides and primers sequences using the filterAndTrim function with the following parameters: truncLen = c(290, 220), trimLeft = c(50, 55), and maxN = 0, truncQ = 2. The amplicon sequence variants were inferred using the DADA2 core sample inference algorithm with default parameters. Forward and reverse reads were merged and reads with mismatches were removed. Chimeras were identified using the removeBimeraDenovo function and removed. Further, the SILVA database release 132 (Quast et al., 2013) was used for the taxonomic assignment. Finally, the AVSs table was rarefied to 25,181 reads per sample.

### Statistical Analysis

Statistical analyses were carried out using Phyloseq 1.32 (McMurdie and Holmes, 2013) and Vegan 2.5 (Dixon, 2003) packages. The differences between the geophagic and non-geophagic control soil composition were tested via Mann–Whitney *U*-test. Alpha diversity was explored considering the Shannon index and Observed richness calculated from the rarefied AVSs table (25,181 reads). Both indices values were checked for normality using the Shapiro–Wilk test. The possible effects of sex, age class, and family group on alpha diversity indices were evaluated with a Linear Model (ANOVA type III). Beta dispersion was calculated to test if the groups, classified according to sex, age class, and family group, had the same centroids and heterogeneity. Permutational multivariate analysis of variance (PERMANOVA) was applied to test the possible effect of sex, age class, and family group on the bacterial communities. In addition, the Constrained Analysis of Principal Coordinates (CAP) based on Bray Curtis was used to generate the ordination plots.

Linear discriminant analysis effect size (LEfSe) algorithm (LDA score ≥ 2 and *p*-value < 0.05) was applied to detect the biomarker taxa for each category (Segata et al., 2011). We excluded from the LEfSe analysis the family groups with less than three individuals (i.e., 4MZ and 6MZ).

## Results

### Geophagy Site Characterization

**FIGURE 2:**
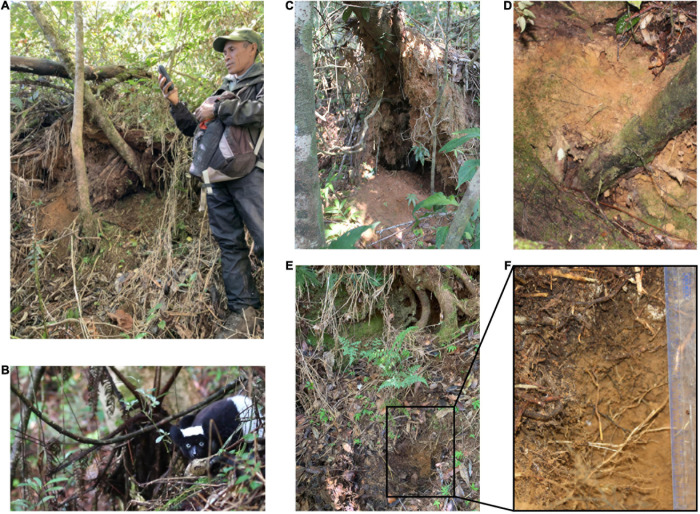
Visualization of **(A)** a research guide recording the location of a geophagic site (GPS waypoint); **(B)** an indri (*Indri indri*) performing geophagic behavior, eating soil in a specific site; **(C)** a geophagic site under a fallen tree; **(D)** soil horizon eaten in the geophagic site; **(E)** a control site with the upper surface untouched; and **(F)** enlargement of the soil sampled in the control site, under the surface, in the horizon normally eaten by indri. Soil is collected free of debris (grass, leaves, stones, roots).

Indris were observed to eat soil in sites at the bases of trees uprooted by wind and/or by rainfall, with the lower soil horizons exposed (Figure 2 and Supplementary Video 1). Geophagic and non-geophagic soil samples were characterized by an acidic pH and rich content in total C and N. With regards to the pseudo-total metals, soil samples showed poor content in Calcium (Ca), Phosphorus (P), Sulfur (S), and higher content in Iron (Fe). Manganese (Mn) and Fe were the most extractable in ammonium nitrate in the case of available metals (Supplementary Table 1).

**FIGURE 3:**
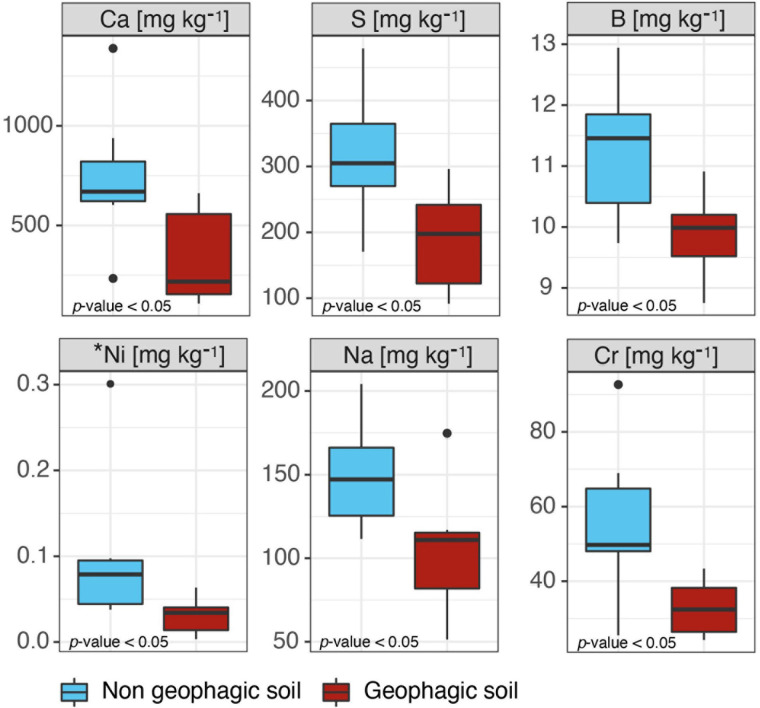
Boxplots representing the chemical parameters resulted statistically different (*p*-value < 0.05) between the geophagic and non-geophagic soils. *Available metal.

Some differences were found between geophagic and non-geophagic sites. Specifically, the concentration of Ca, S, sodium (Na), chromium (Cr), boron (B), and available Nickel (Ni) resulted in being higher in non-geophagic than in geophagic soil samples (*p*-value < 0.01) (Figure 3). On the other hand, for all the other parameters, including pH, total C, total N, the remaining pseudo-total elements, and metals extractable in ammonium nitrate, no statistically significant differences were observed (Supplementary Table 1).

### Bacterial Taxonomic Community Composition

**FIGURE 4:**
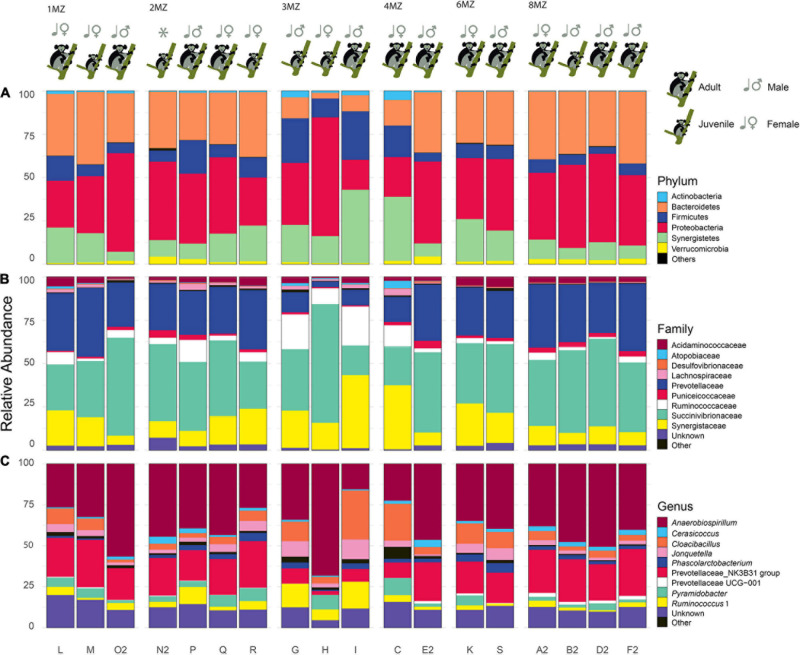
Bar plots of each individual representing the most abundant taxa (average > 1%). Phyum **(A)**, family **(B)**, and genus **(C)**. “Unclassified” represents ASV not classified for the considered taxonomic level. The taxa with a relative average abundance < 1% are collapsed in “Others”. In addition, information regarding family group, class age, and sex is reported on the top of each plot.

After quality checking and filtering, 645,297 reads (including non-bacterial reads) were generated from the MiSeq run. The reads assigned as Bacteria were 616,269 resulting in 131 amplicon sequence variants (Supplementary Tables 2, 3). Rarefaction curves showed that all the samples nearly reached the plateau (Supplementary Figure 1). All the samples were identified at phylum level: Proteobacteria 40.1 ± 9.5%, Bacteroidetes 28.7 ± 2.8%, Synergistetes 16.7 ± 4.5%, Firmicutes 11.1 ± 1.9%, Verrucomicrobia 2.0 ± 1.2%, Actinobacteria 1.2 ± 0.6%, and Cyanobacteria 0.2 ± 0.3% (Figure 4A). At family level the most abundant groups were: Succinivibrionaceae 39.6 ± 11.6%, Prevotellaceae 26.4 ± 3.2%, Synergistaceae 16.7 ± 4.5%, Ruminococcaceae 6.6 ± 2.7%, Acidaminococcaceae 3.3 ± 1.2%, and Puniceicoccaceae 2.0 ± 1.2% (Figure 4B). At a finer taxonomic level, the prevalent genera identified were: *Anaerobiospirillum* 39.3 ± 11.9% and Prevotellaceae NK3B31 group 19.8 ± 3.8%, *Cloacibacillus* 8.2% ± 7.2%, *Ruminococcus* 1, 5.0 ± 2.8%, *Jonquetella* 4.24% ± 2.8%, *Pyramidobacter* 4.0 ± 2.8%, *Phascolarctobacterium* 2.6 ± 1.2%, and *Cerasicoccus* 2.0 ± 1.2% (Figure 4C).

### Effect of Family Group, Sex, and Age Class on Indri Bacterial Diversity

Considering all the individuals, the mean Shannon diversity was 2.61 ± 0.26, whereas the Observed richness’s value was 45 ± 7. The values for each individual are reported in Table 1. Shannon diversity and Observed richness data resulted to be normally distributed (Shapiro–Wilk normality test: Observed richness, *W* = 0.92, *p*-value = 0.14; Shannon diversity, *W* = 0.91, *p*-value = 0.07).

The Linear Model revealed that Observed richness was influenced by family group (*F* = 17.69, *p*-value = 0.0002), whereas Shannon diversity was affected by both family group (*F* = 4.37, *p*-value = 0.02) and sex (*F* = 10.02, *p*-value = 0.01). In particular, females showed higher alpha diversity values if compared to males. Finally, no significant effect was detected according to the age class (Supplementary Table 4).

**FIGURE 5:**
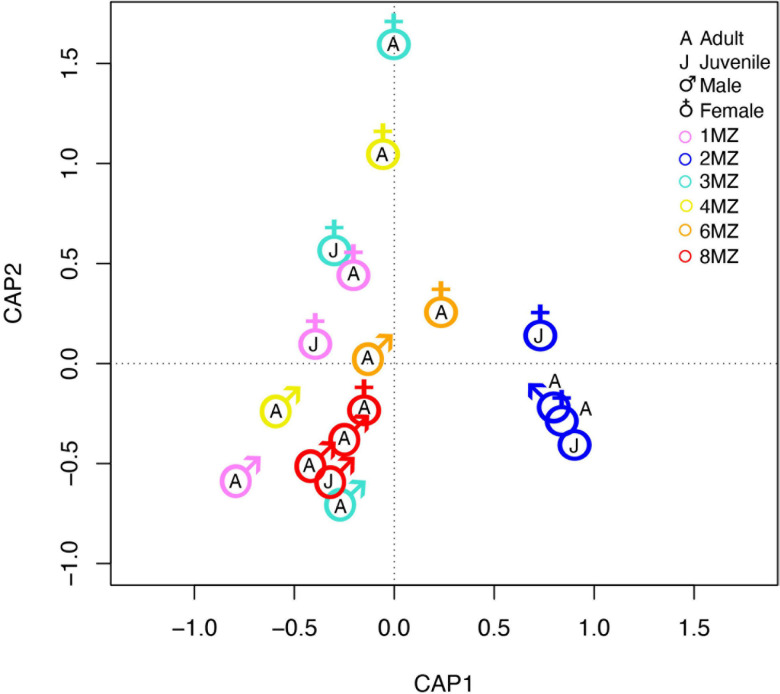
Constrained analysis of principal coordinate ordination plot on bacterial communities of indris fecal samples.

Beta-dispersion of bacterial communities revealed that the samples had homogeneous dispersion (Sex, *F* = 1.24 and *p*-value = 0.31; family group, *F* = 1.21 and *p*-value = 0.43; age class *F* = 0.002 and *p*-value = 0.98). PERMANOVA analysis showed that sex (*F* = 7.43, *p*-value = 0.001) and family group (*F* = 7.4707, *p*-value = 0.001) resulted to significantly affect the bacterial communities’s beta-diversity, differently from age class (*F* = 0.89, *p*-value = 0.51). Further, CAP analysis, confirming the results obtained with the PERMANONVA, found that among all the tested possible drivers, sex, and family group influenced the bacterial community’s structure (com ∼ family group + Sex; *F* = 5.94 *p*-value = 0.001) (Figure 5).

**FIGURE 6:**
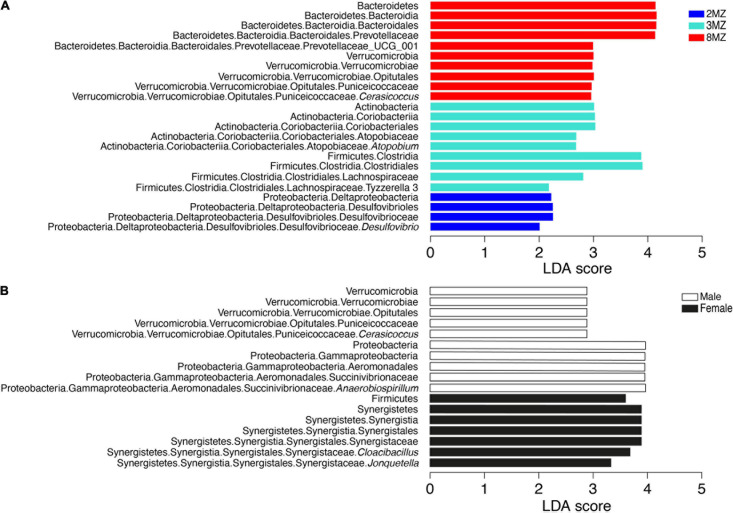
Linear discriminant analysis effect size plots of the biomarkers taxa for the categories family group **(A)** and sex **(B)**.

Linear discriminant analysis effect size algorithm found 15 ASVs biomarkers for the group 1MZ, 17 ASVs with 2MZ, 11 ASVs with 3MZ, and 25 with 8MZ (Supplementary Table 5). At phylotype level, Proteobacteria, mainly with the genus *Desulfovibrio*, characterized the group 2MZ, whereas Actinobacteria with *Atopobium* and Firmicutes with *Tyzzerella* 3 were biomarkers of 3MZ (Figure 6A). Further, Bacteroidetes with Prevotellaceae UCG001 and Verrucomicrobia with *Cerasicoccus* were more abundant in the group 8MZ (Figure 6A).

Concerning sex, four AVSs biomarkers were found for females and two AVSs males (Supplementary Table 6). Moreover, Firmicutes and Synergistetes with the genera *Cloacibacillus* and *Jonquetella* were more abundant in females; differently, Verrucomicrobia with the genus *Cerasicoccus* and Proteobacteria with the genus *Anaerobiospirillum* were mainly present in males (Figure 6B).

## Discussion

### Indris Gut Microbiome Diversity

Although in different proportions, the most abundant phyla found in indris’ gut (i.e., Proteobacteria, Bacteroides, and Firmicutes) are consistent with those found in other studies involving primates (Aivelo et al., 2016). On the other hand, the relative abundance of Proteobacteria found in our study was almost five times higher than that found in other lemurs species, such as *Lemur catta* (Umanets et al., 2018), *Eulemur rufifrons*, and *E. rubriventer* (Bennett et al., 2016; Table 2). Nevertheless, Greene et al. (2020) investigating wild indris’ gut microbiome diversity found a higher abundance of Proteobacteria compared to the other three lemur species (i.e., *L. catta*, *E. rufifrons*, and *E. rubriventer*) (Bennett et al., 2016; Umanets et al., 2018), but still lower than what we found in our work (Table 2). With this regard, the high relative abundance of Proteobacteria present in our samples and found in Greene et al. (2020) could represent the typical composition of the gut microbiome of healthy individuals. Differently, in humans, an increased prevalence of Proteobacteria has been observed as a potential signature of dysbiosis (Illiano et al., 2020). Specifically, altered homeostasis, caused by environmental or host factors, such as a low-fiber diet and acute or chronic inflammation, could be a selection driver and cause dysbiosis with an increased number of Proteobacteria in the gut. For what concerns the indris, their diet is based on fiber due to its folivores’ habitus, with usual consumption of soil as integration. Plant leaves and soil could most likely be an important source of Proteobacteria; in fact, plant leaves, and soil contain about 62 and 36.5% of Proteobacteria, respectively (Shin et al., 2015). Proteobacteria could play a key role in cinnamates degradation and hydroxycinnamates and hydroxycinnamic acids utilization for energy recovery (Greene et al., 2020). Further, indris might rely primarily on Proteobacteria, and secondly on Bacteroidetes and Firmicutes (e.g., *Prevotella* and *Ruminobacter*) for fiber digestion (Biddle et al., 2013). Indeed, Firmicutes members such as Lachnospiraceae and Ruminococcaceae, with some Bacteroidetes, have known fiber fermenting abilities. Interestingly, they have been associated with the production of the appreciated colonocyte nutrient butyrate (Biddle et al., 2013; Meehan and Beiko, 2014). The presence of functionally redundant taxa might support functional stability during ordinary life and possible life disturbance (Vital et al., 2017).

**TABLE 2:** Percentage of the three top bacterial phyla found in this study and other studies.

**Lemurs species**	**References**	**Firmicutes (%)**	**Bacteroidetes (%)**	**Proteobacteria (%)**
*Lemur catta*	Umanets et al., 2018	51.57 ± 0.11	15.81 ± 0.11	5.21 ± 0.11
*Eulemur rufifrons* and *E. rubriventer*	Bennett et al., 2016	43.3 ± 0.064	30.3 ± 0.053	7.4 ± 0.031
*I. indri*	Greene et al., 2020	19.70	47.70	20.50
*I. indri*	This study	11.1 ± 1.9	28.7 ± 2.8	40.1 ± 9.5

Regarding the factors driving microbial diversity, this study showed the crucial role of social groups in shaping the indris microbiome for the first time. Differences among social groups may be related to feeding and social interactions like grooming, which provide close contact between subjects of the same group (Bennett et al., 2016; Raulo et al., 2018). These mechanisms were identified as relevant factors influencing the microbiome composition of baboons and chimpanzees (Degnan et al., 2012; Tung et al., 2015). A study that analyzed the dynamics of the composition of 10 wild groups in the Maromizaha NAP, comprising the groups sampled in this work, found evidence of only one immigrant female and one immigrant male out of 68 indris over 12 years (Rolle et al., 2021 in press). This very low rate of intergroup mobility limits the number of social partners that indri can have in their lives and, consequently, the intergroup transmission of microorganisms and parasites. In addition, sex was another factor that significantly influenced the microbiome alpha and beta-diversity. Particularly, the higher bacterial Shannon diversity found in females than males could be due to the sex hormones that play a crucial role in sex dimorphism (Haro et al., 2016). Moreover, females showed a higher abundance of *Cloacibacillus* and *Jonquetella*, both belonging to the novel phylum Synergistetes, that inhabits the mammalian gastrointestinal tract typically (Jumas-Bilak et al., 2007; Looft et al., 2013). Differently, males had a higher abundance of bacteria from the *Anaerobiospirillum* genus. This difference can be explained by the fact that females and males differ in nutritional and energetic demands for growth, development, and reproduction. Moreover, sex-specific traits influence the ecological structure of the gut microbiome, maintaining sex differences in physiology and behavior throughout life (Jašarević et al., 2016).

### Geophagy in Indris

Typical Oxisols with a reddish color characterized geophagic and non-geophagic sampling sites. Some inherent characteristics of the Oxisols, such as the quite acidic pH, the richness of secondary oxide-hydroxides and highly weathered clays, seem more important for geophagy than the content in pseudo-total or available elements (Vågen et al., 2006; Borruso et al., 2021). According to the adaptive hypothesis of geophagy, the soil ingested by indri could play a crucial role in micronutrient supplementation and detoxification (i.e., adsorption functions via oxyhydroxides and clays) (Pebsworth et al., 2019). Indeed, indris are folivorous, consuming mainly immature leaves rich in potentially toxic compounds such as tannins, terpenes, and cyanogenic glycosides derived (Hemingway, 1998); thus, the geophagic soil could be involved in the plant’s toxin adsorption derived from the diet (de Souza et al., 2002; Pebsworth et al., 2019).

However, the reason behind the selection of one site instead of another one remains unclear. The choice of the sites characterized by the exposition of lower soil horizons could be a strategy to limit the energy expended in obtaining soil from the intact ground. Nevertheless, some elements (i.e., Ca, S, Na, Cr, B, and available Ni) were present at lower concentrations in geophagic than in non-geophagic soil. Although we cannot directly explain these differences, they could indicate that other soil quality traits could orientate the selection of a specific soil.

In conclusion, studies on different species suggested that geophagic sites are required to maintain individual and population health (Pebsworth et al., 2019). Accordingly, preserving the geophagic sites is crucial in wildlife conservation policy.

### Microbial Ecology and Indri Conservation

Microbial ecology offers valuable perspectives to investigate primate health and improve conservation efforts. Understanding the drivers affecting the microbiome associated with the host (e.g., indri) is critical for conservation biology. It is well known that the microbial gut communities profoundly affect host health, nutrition, physiology, and immune systems (Sandri et al., 2020). For instance, our study is fundamental to document the typical composition of healthy individuals considering sex and group influence (Amato et al., 2020). Therefore, many studies have been conducted on the human microbiome where microbial biomarkers of health have been shown, such as the presence of *Faecalibacterium prausnitzii* (Manor et al., 2020). The acquisition of new information about animal gut microbiomes can help identify biomarkers for animal health. In addition, microbial gut communities are sensitive to environmental alterations and their diversity seems to be correlated with habitat quality and, thus, with possible health implications (Scotti et al., 2017). The application of gut microbiome analyses to wildlife conservation of endangered species is currently in its infancy but holds enormous potential. To date, no conservation policy or legislation includes microbiome assessments. Integrating a new understanding of the patterns of microbial diversity and early signs of impending microbial disruption offer valuable tools for informing conservation strategies and monitoring and promoting primate health (Stumpf et al., 2016). The present study represents a first insight toward understanding the overall diversity and ecology of indris microbiome in different familiar groups and a sex-dependent baseline that can be tracked over time as a component of efforts to help animal conservation.

## Data Availability Statement

The datasets presented in this study can be found in online repositories. The names of the repository/repositories and accession number(s) can be found below: Sequence Read Archive (SRA) BioProject ID: PRJNA701813.

## Ethics Statement

Ethical review and approval was not required for the animal study because. The non-invasive methods used for fecal collections of wild indris adhere to the International Primatological Society (IPS) “Principles for the Ethical Treatment of Non-Human Primates.” Field data collection protocols were reviewed and approved by Madagascar’s Ministere de l’Environnement, de l’Ecologie et des Forêts (Permit 2018: N° 91/18/MEEF/SG/DGF/DSAP/SCB.Re). Field data collection protocols were also approved by Groupe d’Étude et de Recherche sur les Primates de Madagascar (GERP), the association governing research in the Maromizaha New Protected Area.

## Author Contributions

CSa CSp, CG, PM, PT, and LB conceived and designed the experiments. VT and RR supervised the collection of the fecal and soil samples. FC, AC, DL, LC, and MM carried out the experiments. FC, LB, DL, MM, VT, LC, MS, TM, AC, MD, and FB analyzed the data. FC, AC, LB, CSa, CSp, CG, VT, SC, TM, and PM wrote the manuscript. All authors contributed to the article and approved the submitted version.

## Conflict of Interest

The authors declare that the research was conducted in the absence of any commercial or financial relationships that could be construed as a potential conflict of interest.

## Publisher’s Note

All claims expressed in this article are solely those of the authors and do not necessarily represent those of their affiliated organizations, or those of the publisher, the editors and the reviewers. Any product that may be evaluated in this article, or claim that may be made by its manufacturer, is not guaranteed or endorsed by the publisher.
