# Supplementary material for: Disentangling the Possible Drivers of Indri indri Microbiome: A Threatened Lemur Species of Madagascar
Source: Front Microbiol. 2021 Aug 6;12:668274. doi: 10.3389/fmicb.2021.668274 (PMC8378179; doi:10.3389/fmicb.2021.668274)

**Disentangling the possible drivers of *Indri indri* microbiome: A threatened lemur species of Madagascar**

Federico Correa^1^, Valeria Torti^2^, Caterina Spiezio^3^, Alice Checcucci^1^, Monica Modesto^1^, Luigimaria Borruso^4^, Luciano Cavani^1^, Tanja Mimmo^4^, Stefano Cesco^4^, Diana Luise^1^, Rose M. Randrianarison ^5,6^, Marco Gamba^2^, Nianja J. Rarojoson^7^, Maurizio Sanguinetti^8,9^,Maura Di Vito^8^, Francesca Bugli^8,9^, Paola Mattarelli^1^, Paolo Trevisi^1^, Cristina Giacoma^2^, Camillo Sandri^1,3^

^1^Department of Agricultural and Food Sciences, University of Bologna, Viale Fanin 44, 40127 Bologna, Italy

^2^Department of Life Sciences and Systems Biology, University of Torino, Torino, Italy.

^3^Department of Animal Health Care and Management, Parco Natura Viva - Garda Zoological Park, Bussolengo, Verona, Italy

^4^Faculty of Science and Technology, Free University of Bolzano-Bozen, Piazza Università 5, 39100 Bolzano-Bozen, Italy

^5^Groupe d’Étude et de Recherche sur les Primates de Madagascar (GERP), Cité des Professeurs – Fort Duchesne, BP 779 – Antananarivo 101 – Madagascar

^6^Mention d'Anthropobiologie et de Développement Durable (MADD), Université de Antananarivo, Madagascar

^7^Laboratoire de Pédologie, FOFIFA à Tsimbazaza, BP.1690 Antananarivo.

^8^Dipartimento di Scienze Biotecnologiche di Base, Cliniche Intensivologiche e Perioperatorie, Università Cattolica del Sacro Cuore, Largo A. Gemelli 8, 00168 Rome, Italy;

^9^Dipartimento di Scienze di Laboratorio e Infettivologiche, Fondazione Policlinico Universitario A. Gemelli IRCCS, Largo A. Gemelli 8, 00168 Rome, Italy

* **Correspondence:**

Luigimaria Borruso

[luigimaria.borruso@unibz.it](mailto:luigimaria.borruso@unibz.it)

Paola Mattarelli

paola.mattarelli@unibo.it

**Contents**

1. Supplementary Tables 6
2. Supplementary Figures 1

**1. SUPPLEMENTARY TABLES**

**Table S1.** Geophagic and non-geophagic soil characteristics (average ± standard error) and Mann–Whitney test *p*-value (n.s. = not significant for *p*-value > 0.05, DL = detection limit)

| Parameter | Unit | Non-geophagic soil | Geophagic soil | *p*-value |
| --- | --- | --- | --- | --- |
| pH |  | 4.17 ± 0.14 | 4.51 ± 0.13 | n.s. |
| Total N | % | 0.17 ± 0.03 | 0.22 ± 0.04 | n.s. |
| Total C | % | 2.40 ± 0.42 | 3.14 ± 0.54 | n.s. |
| C/N ratio |  | 14.3± 0.6 | 14.1 ± 0.7 | n.s. |
| Pseudo total elements: |  |  |  |  |
| Al | mg kg^-1^ | 72200 ± 3280 | 71300 ± 6870 | n.s. |
| As | mg kg^-1^ | 0.346 ± 0.223 | 1.001 ± 0.392 | n.s |
| B | mg kg^-1^ | 11.2 ± 0.4 | 9.8 ± 0.3 | 0.03 |
| Ba | mg kg^-1^ | 18.5 ± 4.6 | 28.0 ± 8.6 | n.s. |
| Be | mg kg^-1^ | 0.615 ± 0.164 | 0.760 ± 0.148 | n.s. |
| Ca | mg kg^-1^ | 739 ± 133 | 344 ± 89 | 0.01 |
| Co | mg kg^-1^ | 4.70 ± 1.18 | 5.85 ± 1.49 | n.s |
| Cr | mg kg^-1^ | 56.2 ± 7.9 | 32.7 ± 2.8 | 0.02 |
| Cu | mg kg^-1^ | 12.5 ± 4.7 | 9.9 ± 2.1 | n.s |
| Fe | g kg^-1^ | 28.7 ± 2.6 | 32.4 ± 5.4 | n.s |
| K | mg kg^-1^ | 670 ± 219 | 840 ± 308 | n.s |
| Li | mg kg^-1^ | 3.63 ± 1.11 | 4.85 ± 1.52 | n.s |
| Mg | mg kg^-1^ | 353 ± 113 | 267 ± 75 | n.s |
| Mn | mg kg^-1^ | 120 ± 37 | 199 ± 54 | n.s |
| Mo | mg kg^-1^ | 2.86 ± 0.59 | 2.66 ± 0.57 | n.s |
| Na | mg kg^-1^ | 149 ± 12 | 104 ± 15 | 0.04 |
| Ni | mg kg^-1^ | 15.70 ± 5.92 | 10.71 ± 3.55 | n.s |
| P | mg kg^-1^ | 229 ± 34 | 243 ± 41 | n.s |
| Pb | mg kg^-1^ | 34.1 ± 3.1 | 49.4 ± 13.1 | n.s |
| S | mg kg^-1^ | 317 ± 37 | 187 ± 30 | 0.02 |
| Si | mg kg^-1^ | 302 ± 147 | 300 ± 93 | n.s |
| Sn | mg kg^-1^ | 2.70 ± 0.36 | 2.96 ± 0.34 | n.s |
| Sr | mg kg^-1^ | 10.67 ± 2.09 | 5.43 ± 1.12 | n.s |
| Ti | mg kg^-1^ | 1771 ± 139 | 1804 ± 398 | n.s |
| V | mg kg^-1^ | 34.4 ± 9.9 | 41.5 ± 19.7 | n.s |
| Zn | mg kg^-1^ | 54.9 ± 7.6 | 57.9 ± 10.3 | n.s |
| Available metals: |  |  |  |  |
| Cd | mg kg^-1^ | < DL | < DL | n.s |
| Co | mg kg^-1^ | < DL | < DL | n.s |
| Cr | mg kg^-1^ | < DL | < DL | n.s |
| Cu | mg kg^-1^ | 0.023 ± 0.010 | 0.022 ± 0.008 | n.s |
| Fe | mg kg^-1^ | 8.64 ± 4.13 | 6.00 ± 2.27 | n.s |
| Mn | mg kg^-1^ | 8.91 ± 3.73 | 3.40 ± 1.28 | n.s |
| Ni | mg kg^-1^ | 0.099 ± 0.03 | 0.030 ± 0.011 | 0.015 |
| Pb | mg kg^-1^ | 0.584 ± 0.138 | 3.167 ± 1.197 | n.s |
| Zn | mg kg^-1^ | 0.712 ± 0.221 | 0.425 ± 0.161 | n.s |

**Table S2**: Bacterial sequences obtained per each sample after filtering.

| Sample | Bacterial reads |
| --- | --- |
| A2 | 48,119 |
| B2 | 36,307 |
| C | 27,177 |
| D2 | 38,312 |
| E2 | 28,294 |
| F2 | 45,553 |
| G | 26,102 |
| H | 34,972 |
| I | 27,262 |
| K | 37,193 |
| L | 39,381 |
| M | 25,181 |
| N2 | 31,,619 |
| O2 | 34,686 |
| P | 32,653 |
| Q | 32,819 |
| R | 35,349 |
| S | 35,242 |

**Table S3**: Statistic summary of the overall bacterial sequences.

| Bacterial reads summary | |
| --- | --- |
| Total count | 616,221 |
| Min | 25,181 |
| Max | 48,119 |
| Median | 34,829 |
| Mean | 34,235 |
| Std. dev. | 6,293 |

**Table S4.** Linear model of the Observed richness and Shannon indices tested for the categories: family group, sex and age.

| Alpha diversity linear model | | | | | |
| --- | --- | --- | --- | --- | --- |
| Observed richenss | Sum. Sq. | Df | F value | *p*-value |  |
| (Intercept) | 4249.0 | 1 | 496.34 | 3.499e-09 | *** |
| Group | 757.3 | 5 | 17.69 | 0.0002 | *** |
| Sex | 0.4 | 1 | 0.04 | 0.84 |  |
| Class age | 0.2 | 1 | 0.02 | 0.88 |  |
| Residuals | 77.0 | 9 |  |  |  |
| Shannon | Sum. Sq. | Df | F value | *p*-value |  |
| (Intercept) | 15.8816 | 1 | 488.58 | 3.751e-09 | *** |
| Group | 0.7111 | 5 | 4.37 | 0.02 | * |
| Sex | 0.3259 | 1 | 10.02 | 0.01 | * |
| Class age | 0.0026 | 1 | 0.08 | 0.78 |  |
| Residuals | 0.2925 | 9 |  |  |  |

**Table S5.** LEfSe analysis identification of the biomarker taxa for the category family groups.

| Taxonomy |  | LDA | *p*-value |
| --- | --- | --- | --- |
| Bacteria.Actinobacteria | 3MZ | 3.01 | 0.0186 |
| Bacteria.Actinobacteria.Coriobacteriia | 3MZ | 3.03 | 0.0186 |
| Bacteria.Actinobacteria.Coriobacteriia.Coriobacteriales | 3MZ | 3.03 | 0.0186 |
| Bacteria.Actinobacteria.Coriobacteriia.Coriobacteriales.Atopobiaceae | 3MZ | 2.68 | 0.0112 |
| Bacteria.Actinobacteria.Coriobacteriia.Coriobacteriales.Atopobiaceae.*Atopobium* | 3MZ | 2.68 | 0.0112 |
| Bacteria.Actinobacteria.Coriobacteriia.Coriobacteriales.Atopobiaceae.*Atopobium*.ASV49 | 3MZ | 2.70 | 0.0112 |
| Bacteria.Actinobacteria.Coriobacteriia.Coriobacteriales.Coriobacteriaceae.*Collinsella*.ASV390 | 3MZ | 2.33 | 0.0051 |
| Bacteria.Bacteroidetes | 8MZ | 4.14 | 0.0496 |
| Bacteria.Bacteroidetes.Bacteroidia | 8MZ | 4.16 | 0.0496 |
| Bacteria.Bacteroidetes.Bacteroidia.Bacteroidales | 8MZ | 4.16 | 0.0290 |
| Bacteria.Bacteroidetes.Bacteroidia.Bacteroidales.Prevotellaceae | 8MZ | 4.14 | 0.0439 |
| Bacteria.Bacteroidetes.Bacteroidia.Bacteroidales.Prevotellaceae.Prevotellaceae_NK3B31_group.ASV105 | 2MZ | 2.72 | 0.0334 |
| Bacteria.Bacteroidetes.Bacteroidia.Bacteroidales.Prevotellaceae.Prevotellaceae_NK3B31_group.ASV106 | 8MZ | 2.74 | 0.0057 |
| Bacteria.Bacteroidetes.Bacteroidia.Bacteroidales.Prevotellaceae.Prevotellaceae_NK3B31_group.ASV11 | 1MZ | 3.57 | 0.0155 |
| Bacteria.Bacteroidetes.Bacteroidia.Bacteroidales.Prevotellaceae.Prevotellaceae_NK3B31_group.ASV12 | 2MZ | 3.76 | 0.0057 |
| Bacteria.Bacteroidetes.Bacteroidia.Bacteroidales.Prevotellaceae.Prevotellaceae_NK3B31_group.ASV13 | 8MZ | 3.63 | 0.0057 |
| Bacteria.Bacteroidetes.Bacteroidia.Bacteroidales.Prevotellaceae.Prevotellaceae_NK3B31_group.ASV14 | 2MZ | 3.47 | 0.0271 |
| Bacteria.Bacteroidetes.Bacteroidia.Bacteroidales.Prevotellaceae.Prevotellaceae_NK3B31_group.ASV18 | 8MZ | 3.45 | 0.0101 |
| Bacteria.Bacteroidetes.Bacteroidia.Bacteroidales.Prevotellaceae.Prevotellaceae_NK3B31_group.ASV213 | 8MZ | 2.41 | 0.0334 |
| Bacteria.Bacteroidetes.Bacteroidia.Bacteroidales.Prevotellaceae.Prevotellaceae_NK3B31_group.ASV242 | 2MZ | 2.44 | 0.0057 |
| Bacteria.Bacteroidetes.Bacteroidia.Bacteroidales.Prevotellaceae.Prevotellaceae_NK3B31_group.ASV264 | 8MZ | 2.39 | 0.0057 |
| Bacteria.Bacteroidetes.Bacteroidia.Bacteroidales.Prevotellaceae.Prevotellaceae_NK3B31_group.ASV280 | 8MZ | 2.30 | 0.0167 |
| Bacteria.Bacteroidetes.Bacteroidia.Bacteroidales.Prevotellaceae.Prevotellaceae_NK3B31_group.ASV4 | 1MZ | 3.87 | 0.0072 |
| Bacteria.Bacteroidetes.Bacteroidia.Bacteroidales.Prevotellaceae.Prevotellaceae_NK3B31_group.ASV52 | 3MZ | 3.08 | 0.0482 |
| Bacteria.Bacteroidetes.Bacteroidia.Bacteroidales.Prevotellaceae.Prevotellaceae_NK3B31_group.ASV56 | 8MZ | 2.83 | 0.0057 |
| Bacteria.Bacteroidetes.Bacteroidia.Bacteroidales.Prevotellaceae.Prevotellaceae_NK3B31_group.ASV674 | 8MZ | 2.01 | 0.0334 |
| Bacteria.Bacteroidetes.Bacteroidia.Bacteroidales.Prevotellaceae.Prevotellaceae_UCG_001 | 8MZ | 2.99 | 0.0091 |
| Bacteria.Bacteroidetes.Bacteroidia.Bacteroidales.Prevotellaceae.Prevotellaceae_UCG_001.ASV173 | 1MZ | 2.40 | 0.0082 |
| Bacteria.Bacteroidetes.Bacteroidia.Bacteroidales.Prevotellaceae.Prevotellaceae_UCG_001.ASV182 | 8MZ | 2.36 | 0.0057 |
| Bacteria.Bacteroidetes.Bacteroidia.Bacteroidales.Prevotellaceae.Prevotellaceae_UCG_001.ASV246 | 8MZ | 2.39 | 0.0057 |
| Bacteria.Bacteroidetes.Bacteroidia.Bacteroidales.Prevotellaceae.Prevotellaceae_UCG_001.ASV541 | 8MZ | 2.13 | 0.0057 |
| Bacteria.Bacteroidetes.Bacteroidia.Bacteroidales.Prevotellaceae.Prevotellaceae_UCG_001.ASV59 | 8MZ | 2.61 | 0.0072 |
| Bacteria.Bacteroidetes.Bacteroidia.Bacteroidales.Prevotellaceae.ASV15 | 1MZ | 3.35 | 0.0082 |
| Bacteria.Bacteroidetes.Bacteroidia.Bacteroidales.Prevotellaceae.ASV167 | 1MZ | 2.65 | 0.0051 |
| Bacteria.Bacteroidetes.Bacteroidia.Bacteroidales.Prevotellaceae.ASV20 | 2MZ | 3.41 | 0.0095 |
| Bacteria.Bacteroidetes.Bacteroidia.Bacteroidales.Prevotellaceae.ASV311 | 1MZ | 2.47 | 0.0051 |
| Bacteria.Bacteroidetes.Bacteroidia.Bacteroidales.Prevotellaceae.ASV33 | 8MZ | 3.05 | 0.0057 |
| Bacteria.Bacteroidetes.Bacteroidia.Bacteroidales.Prevotellaceae.ASV36 | 1MZ | 3.06 | 0.0070 |
| Bacteria.Bacteroidetes.Bacteroidia.Bacteroidales.Prevotellaceae.ASV371 | 2MZ | 2.08 | 0.0057 |
| Bacteria.Bacteroidetes.Bacteroidia.Bacteroidales.Prevotellaceae.ASV412 | 1MZ | 2.35 | 0.0051 |
| Bacteria.Bacteroidetes.Bacteroidia.Bacteroidales.Prevotellaceae.ASV74 | 8MZ | 2.48 | 0.0081 |
| Bacteria.Bacteroidetes.Bacteroidia.Bacteroidales.ASV1127 | 1MZ | 2.47 | 0.0051 |
| Bacteria.Bacteroidetes.Bacteroidia.Bacteroidales.ASV146 | 2MZ | 2.64 | 0.0057 |
| Bacteria.Bacteroidetes.Bacteroidia.Bacteroidales.ASV175 | 2MZ | 2.58 | 0.0057 |
| Bacteria.Bacteroidetes.Bacteroidia.Bacteroidales.ASV204 | 3MZ | 2.01 | 0.0482 |
| Bacteria.Bacteroidetes.Bacteroidia.Bacteroidales.ASV248 | 8MZ | 2.50 | 0.0057 |
| Bacteria.Bacteroidetes.Bacteroidia.Bacteroidales.ASV316 | 8MZ | 2.36 | 0.0057 |
| Bacteria.Bacteroidetes.Bacteroidia.Bacteroidales.ASV350 | 8MZ | 2.22 | 0.0057 |
| Bacteria.Bacteroidetes.Bacteroidia.Bacteroidales.ASV351 | 1MZ | 2.46 | 0.0051 |
| Bacteria.Bacteroidetes.Bacteroidia.Bacteroidales.ASV359 | 8MZ | 2.27 | 0.0057 |
| Bacteria.Bacteroidetes.Bacteroidia.Bacteroidales.ASV363 | 8MZ | 2.37 | 0.0057 |
| Bacteria.Bacteroidetes.Bacteroidia.Bacteroidales.ASV393 | 8MZ | 2.41 | 0.0057 |
| Bacteria.Bacteroidetes.Bacteroidia.Bacteroidales.ASV394 | 8MZ | 2.59 | 0.0057 |
| Bacteria.Bacteroidetes.Bacteroidia.Bacteroidales.ASV46 | 1MZ | 2.75 | 0.0106 |
| Bacteria.Bacteroidetes.Bacteroidia.Bacteroidales.ASV474 | 1MZ | 2.16 | 0.0051 |

**Table S6.** LEfSe analysis identification of the biomarker taxa for the category sex.

| Taxonomy | Gender | LDA | p value |
| --- | --- | --- | --- |
| Synergistetes.Synergistia.Synergistales.Synergistaceae.*Jonquetella* | Female | 3.32 | 0.0053 |
| Synergistetes.Synergistia.Synergistales.Synergistaceae | Female | 3.89 | 0.0021 |
| Synergistetes.Synergistia.Synergistales | Female | 3.89 | 0.0021 |
| Synergistetes.Synergistia.Synergistales.Synergistaceae.*Cloacibacillus*.ASV3 | Female | 3.67 | 0.0029 |
| Firmicutes | Female | 3.59 | 0.0269 |
| Synergistetes | Female | 3.89 | 0.0021 |
| Synergistetes.Synergistia.Synergistales.Synergistaceae.*Jonquetella*.ASV7 | Female | 3.31 | 0.0124 |
| Synergistetes.Synergistia.Synergistales.Synergistaceae.ASV130 | Female | 3.71 | 0.0021 |
| Synergistetes.Synergistia | Female | 3.89 | 0.0021 |
| Synergistetes.Synergistia.Synergistales.Synergistaceae.Cloacibacillus | Female | 3.68 | 0.0039 |
| Actinobacteria.Coriobacteriia.Coriobacteriales.ASV331 | Female | 3.41 | 0.0427 |
| Verrucomicrobia.Verrucomicrobiae.Opitutales.Puniceicoccaceae.*Cerasicoccus* | Male | 2.89 | 0.0343 |
| Proteobacteria.Gammaproteobacteria | Male | 3.96 | 0.0015 |
| Verrucomicrobia | Male | 2.89 | 0.0343 |
| Proteobacteria.Gammaproteobacteria.Aeromonadales.Succinivibrionaceae.Anaerobiospirillum | Male | 3.96 | 0.0015 |
| Proteobacteria | Male | 3.96 | 0.0015 |
| Verrucomicrobia.Verrucomicrobiae.Opitutales.Puniceicoccaceae | Male | 2.89 | 0.0343 |
| Verrucomicrobia.Verrucomicrobiae | Male | 2.89 | 0.0343 |
| Proteobacteria.Gammaproteobacteria.Aeromonadales.Succinivibrionaceae | Male | 3.95 | 0.0015 |
| Verrucomicrobia.Verrucomicrobiae.Opitutales.Puniceicoccaceae.*Cerasicoccus*.ASV16 | Male | 2.89 | 0.0433 |
| Proteobacteria.Gammaproteobacteria.Aeromonadales.Succinivibrionaceae.*Anaerobiospirillum*.ASV1 | Male | 3.92 | 0.0206 |
| Verrucomicrobia.Verrucomicrobiae.Opitutales | Male | 2.89 | 0.0343 |
| Proteobacteria.Gammaproteobacteria.Aeromonadales | Male | 3.95 | 0.0015 |

**Figure S1.** Rarefaction curves of the indris’ fecal samples


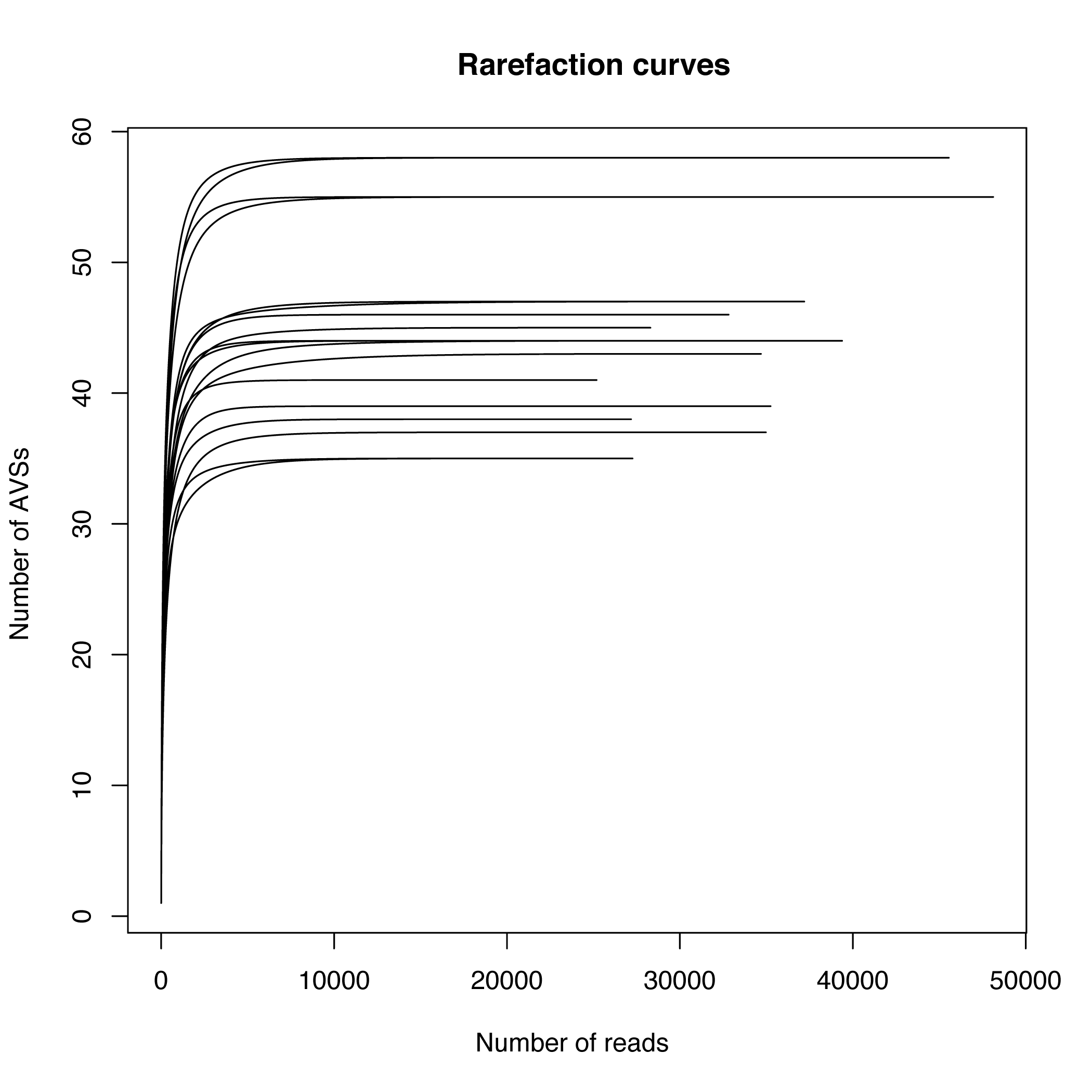

Supplement: Supplementary file 2 [file Data_Sheet_1.docx]
